# Effect of Hydrogen and Absence of Passive Layer on Corrosive Properties of Aluminum Alloys

**DOI:** 10.3390/ma13071580

**Published:** 2020-03-30

**Authors:** Paweł P. Włodarczyk, Barbara Włodarczyk

**Affiliations:** Institute of Environmental Engineering and Biotechnology, Faculty of Natural Sciences and Technology, University of Opole, Kominka Str. 6, 45-032 Opole, Poland; barbara.wlodarczyk@uni.opole.pl

**Keywords:** aluminum alloys, hydrogen, corrosion, reliability, environmental engineering

## Abstract

This paper reports the results of research on the effect of hydrogen permeation and the absence of passive layers on the variations in the corrosive properties of aluminum alloys. The study demonstrated that such variations contribute to the deterioration of corrosive properties, which in turn contributes to shortening the reliability time associated with the operation of aluminum alloy structures. The analysis involved structural aluminum alloys: EN AW-1050A, EN AW-5754, and EN AW-6060. It was demonstrated that the absorption of hydrogen by the analyzed alloys led to the shift of the electrode potential to the negative side. The built hydrogen corrosion cells demonstrate in each case the formation of electromotive force (EMF) cells. The initial EMF value of the cell and its duration depends on the duration of hydrogenation. As a result of removing the passive layers, the electrode potential also changes to the negative side. Following the removal of the passive layer from one of the electrodes, the cells also generated a galvanic (metal) cell. The duration of such a cell is equivalent to the time of restoration of the passive layer. The formation of such hydrogen and metal galvanic cells changes the electrochemical properties of aluminum alloys, therefore deteriorates the corrosive properties of aluminum alloys.

## 1. Introduction

One of the reasons for the degradation of metals and their alloys, both in terms of mechanical and corrosion resistance, is associated with the permeation of hydrogen into them [1,2,3,4,5,6,7]. That situation is particularly important in the case of structural elements (e.g., during their operation), as well as during the transport or production of hydrogen (e.g., hydrogen pipes, fuel cells, pumps, or generators). Hydrogen mainly affects constantly, and in this respect this phenomenon is well-explored, and taken into account during the processes that involve them [1,2,3,5,6,7]. However, aluminum alloys have been increasingly applied in recent decades [8,9,10,11,12,13,14,15]. They are employed in boat and ship designs, in aviation, astronautics, and in automotive industries, as well as in bridge structures and many other applications. It is very important, therefore, to analyze the effect of hydrogen on these alloys [16,17,18,19,20,21,22,23,24,25].

**Figure 1:**
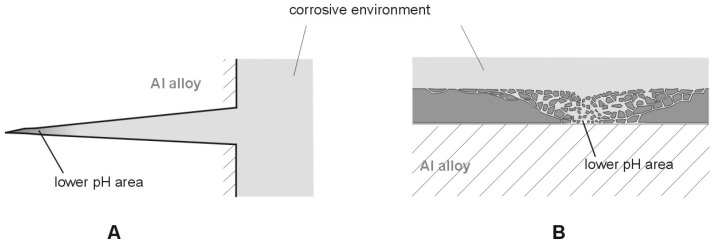
Spots corresponding to greater hydrogen concentrations i.e., lower pH. (Figures are not in scale.) (**A**) Stress corrosion cracking, (**B**) faults in protective coating.

In enclosed or emfi-enclosed areas, when the structure contacts the corrosive solution, the pH of the solution decreases. Such areas can take the form of corrosive cracks (Figure 1A) or originate in a failure of the anti-corrosive coating (Figure 1B). This leads to the hydrogenation of the alloy [26,27,28,29,30,31,32,33,34,35,36,37,38].

**Figure 2:**
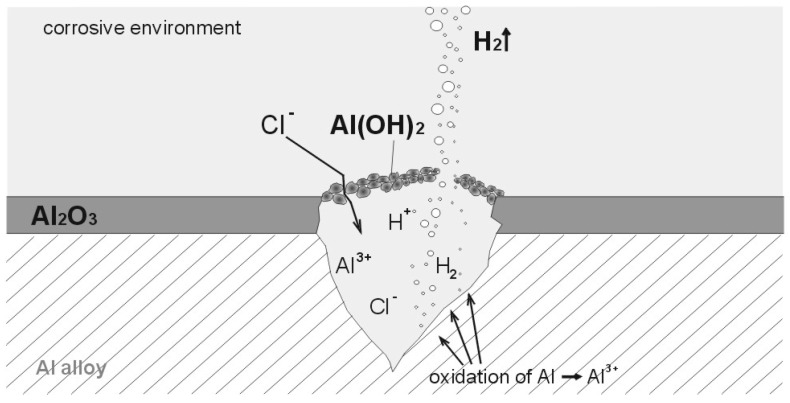
Diagram of pitting on passivated aluminum surface.

Figure 2 presents a pitting on the passivated surface of the aluminum alloy and the location represented by hydrogen formation [27].

**Figure 3:**
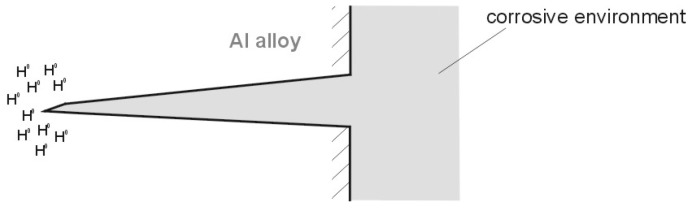
Stress corrosion cracking—distribution of hydrogen absorbed by the metal (or metal alloy).

Hydrogen permeation from solutions is due to the reduction of hydrogen derived from the solution during the cathodic process [20,26,27]. During the cathodic reaction on the metal surface, as a result of the diffusion and migration of the H^+^⋅H_2_O ion, hydrogen cation is reduced, and this is coupled with the formation of H^0^ atoms adsorbed on the surface. Most of the monatomic hydrogen (H^0^) atoms are combined into H_2_ molecules and released into the atmosphere. However, a small amount is absorbed into the metal. This absorption takes place in accordance with laws of diffusion (Fick’s first and second law) [30,31]. The hydrogen permeation in the form of H^0^ is promoted by the very small size of the hydrogen atom compared to the crystal metal structure (or metal alloy). Nevertheless, the distribution of hydrogen over the volume of the metal surface is not uniform. In the places where the highest tensile mechanical stresses occur and the crystal structure expands, the concentration of hydrogen is much higher than the concentration in those elements of the metal structure where tensile stresses are absent. Under mechanical loads, hydrogen grates into the vertices of the stress corrosion cracking gaps (where tensile mechanical loads occur) as a result of long periods of storage, and this considerably accelerates the development of the cracking gaps. Figure 3 illustrates stress cracking with absorbed hydrogen. Hydrogen concentrates in the places of the largest tensile stress [20,26,30,31].

A proportion of the absorbed hydrogen H^0^ accumulates in the metal pores, where it is combined into molecular hydrogen H_2_. If the cathode activity stops, i.e., the process of hydrogen reduction on the metal surface is ceased (e.g., after the metal is removed from a solution or the current flow is discontinued), the hydrogen surface concentration drops to zero, and immediately the process of hydrogen desorption is initiated in the way of diffusion from the metal volume. Hydrogen is removed from the metal throughout the time, lasting approximately one day [31].

In an acidic environment, hydrogen is released from H^+^⋅H_2_O ions, whereas in alkaline solutions it forms H_2_O. The hydrogen release reaction from neutral environments is also possible, but this requires a high potential. It is generally believed that the permeation of hydrogen released from acidic solutions occurs more intensely in comparison to the case when hydrogen is removed from alkaline solutions. Hydrogen permeation into the metal is also intensified by the high concentration of e.g., either arsenic or sulfur in the solution [20,26]. Aluminum alloys are characterized by rapid passivation of the surface, which protects them from susceptibility to the environmental conditions. The failure of a passive layer can have a significant effect on the service life of the structure or equipment. Damage can also occur under mechanical forces acting during operation. The increasing use of aluminum alloys makes it necessary to undertake research concerned with the effect of hydrogen and the deficiencies in passive layers on the variations in the corrosive properties of these alloys. The knowledge of these mechanisms will offer a way in which protection methods can be developed with the purposes of extending the service life and reliability of the structures. From an environmental engineering point of view, the option of extending service lives plays an important practical design consideration. The time needed to produce new components decreases as well, and the material and energy consumption is reduced, as well as the amount of the required maintenance [16,17,20,26]. In addition, when using aluminum alloys in e.g., hydrogen installations (in a fuel cells (FC), in a fuel cell vehicle (FCV), or in a hydrogen generators e.g., in a electrolysers, a bio-hydrogen production installations, or in a microbial electrolysis cells (MEC)), an important consideration is the effect of hydrogen on the materials that are applied in a specific structure [32,33,34,35,36,37,38]. Moreover, the aluminum and aluminum alloys can be used as source of hydrogen [39,40].

This work reports the results of a study concerned with the effect of hydrogen and the absence of passive layers on the electrochemical properties of aluminum alloys, therefore the effect of hydrogen and the absence of passive layers on the deteriorates and the corrosive resistance of aluminum alloys.

## 2. Materials and Methods

The research material included samples of technical aluminum alloys EN AW-1050A, EN AW-5754, and EN AW-6060 applied for plastic reworking [41]. Selected alloys are easily accessible and widely employed as construction materials. All of the analyzed alloys are also easily weldable. The chemical composition of the tested samples is given in Table 1.

**Figure 4:**
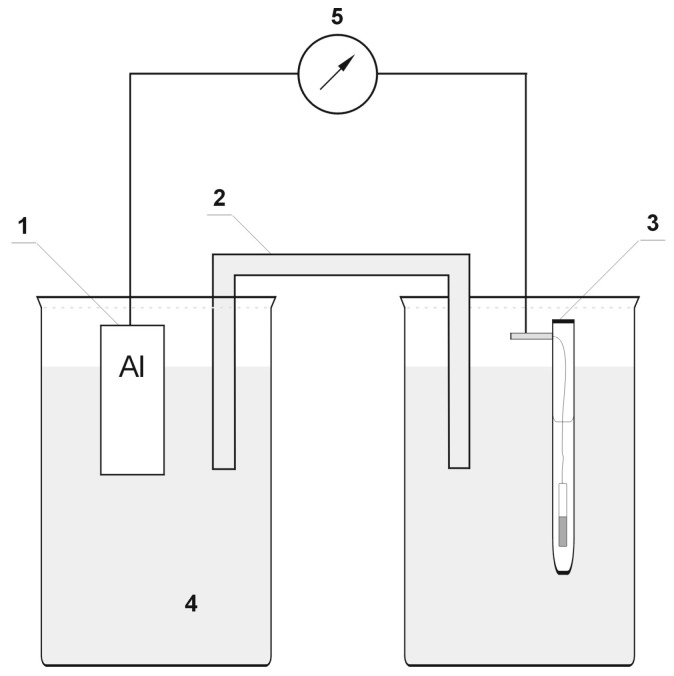
Experimental setup applied in the measurements of electrode potential following hydrogenation (or after the removal of passive layers). 1: sample of alloy, 2: salt bridge, 3: calomel half-cell, 4: electrolyte, 5: multimeter/potentiostat.

With the purpose of assessing whether hydrogen (or the absence of a passive layer) affects the change in alloy surface properties at all, measurements of the variations in in the electrode potential of the investigated alloy following hydrogenation (or after removal of the passive layers) were performed in the initial phase. Figure 4 presents the experimental setup applied for measurement of electrode potential changes.

The calomel half-cell was applied as the reference electrode [42]. The electrolyte was a 3% NaCl solution [18,19,27,32,43]. The samples of alloys for measurements were made as electrodes with the dimensions of 10 × 30 × 1 mm. For shaping, the samples were formed from the analyzed alloys, and they were annealed in a muffle furnace (ELIOG Industrieofenbau GmbH, Römhild, Germany) at a temperature of 2/3 of the melting temperature of aluminum. This procedure was designed to remove internal stresses in the samples.

The hydrogenation of the samples was carried out in a Hoffman apparatus (Fabryka Pomocy Naukowych, Nysa, Poland) in a 0.1N H_2_SO_4_ solution. The anode was made of platinum, whereas the alloys formed the cathode in the process. The surface area of both electrodes was identical. The samples (analyzed alloys) were hydrogenated for 5, 15, and 25 minutes. Cathodic polarization was carried out at a current of 20 mA. On the other hand, the removal of passive layers was carried out mechanically using fine-grained sandpaper.

**Figure 5:**
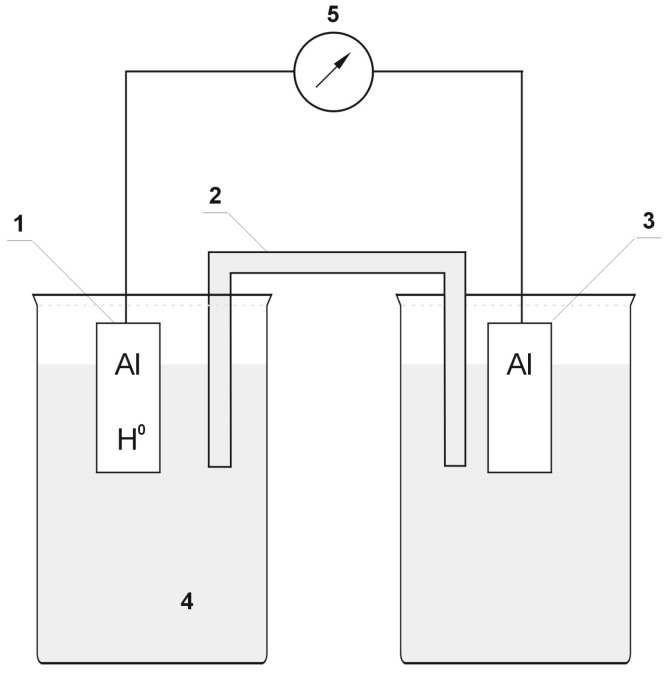
Galvanic cell for measuring the variations in electromotive force (EMF) after hydrogenation (or after removing passive layers). 1: hydrogenated (or without passive layers) sample of alloy, 2: salt bridge, 3: non hydrogenated (or with passive layers) sample of alloy, 4: electrolyte, 5: multimeter/potentiostat.

In order to investigate the effect of hydrogen (or the absence of the passive layer) on the analyzed aluminum alloys, a galvanic cell was designed in which one of the electrodes was hydrogenated (or did not include passive layers). The difference in potentials of a hydrogenated electrode (or without passive layers) and an unreacted electrode (with passive layers) resulted in the formation of electromotive force (EMF) in the cell. Figure 5 contains the image of the galvanic cell applied for measuring the electromotive force following hydrogenation (or after removing the passive layers) of one electrode. A 3% NaCl solution played the role of the electrolyte. The samples had the dimensions of 10 × 30 × 1 mm.

**Figure 6:**
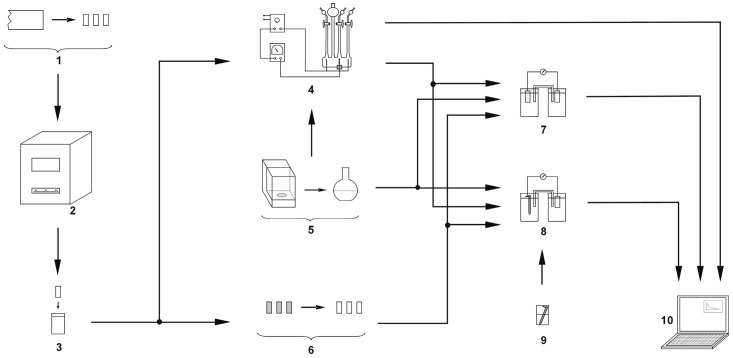
Preparation and experimental setups and schedule of measurements. 1: preparation of alloy samples, 2: annealing in a muffle furnace, 3: degreasing of alloy samples, 4: Hoffman apparatus, 5: electrolyte preparation, 6: removal of passive layers, 7: measurements of electrode potential after hydrogenation (or after removing passive layers), 8: measurements of cell electromotive force (EMF) after hydrogenation (or after removing passive layers), 9: preparation of calomel half-cell, 10: computer.

Figure 6 contains details of the measurement plan: preparation and measuring setup and the order of measurements.

The KS 520/14 silt furnace (ELIOG Industrieofenbau GmbH, Römhild, Germany) was used for annealing of alloy samples. An AMEL System 500 potentiostat (Figure 18; 6) (Amel S.l.r., Milano, Italy) and a Fluke 8840A multimeter (Fluke Corporation, Everett, WA, USA) was used for the electrical measurements. The potentiostat was controlled by a computer with CorrWare software (Scribner Associates Inc., Southern Pines, NC, USA). A Zortrax M200 printer (Zortrax S.A, Olsztyn, Poland) was used to print an electrodes support elements. To prepare the 3D object (the support elements) for printing, Z-Suite software (Zortrax S.A, Olsztyn, Poland) was used. 

## 3. Results

**Figure 7:**
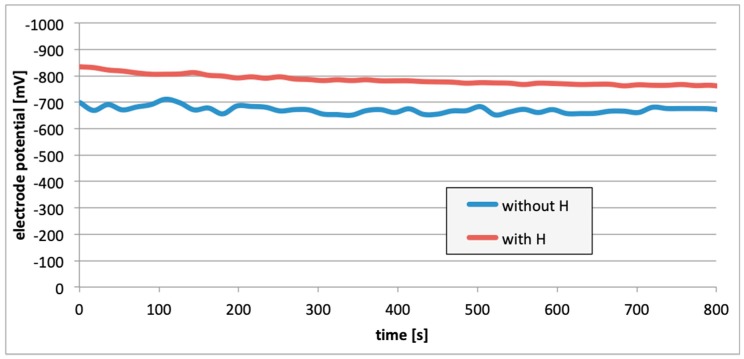
Electrode potential change after the alloy hydrogenation (EN AW-1050A).

**Figure 8:**
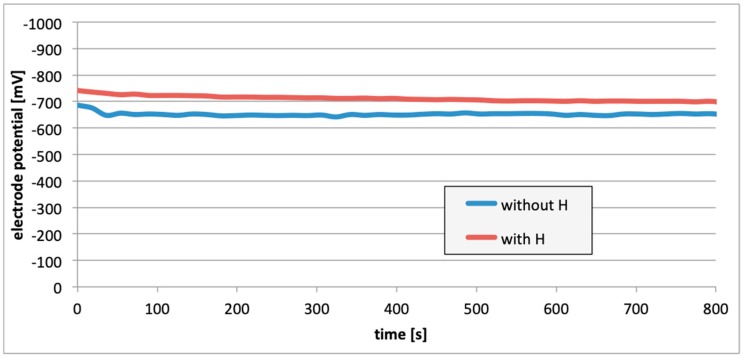
Electrode potential change after the alloy hydrogenation (EN AW-5754).

**Figure 9:**
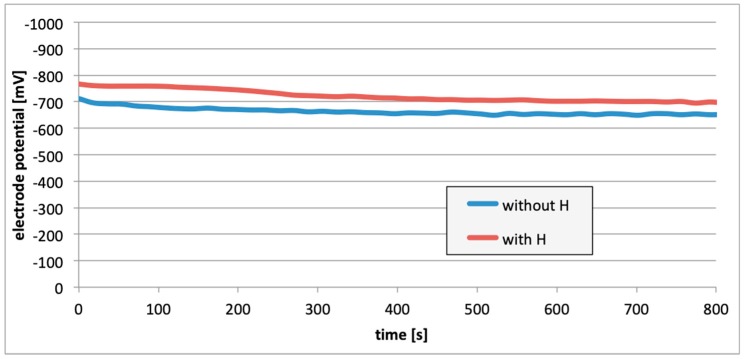
Electrode potential change after the alloy hydrogenation (EN AW-6060).

The entire measurement program was carried out in accordance with the program of measurements presented in Figure 6. Figure 7, Figure 8 and Figure 9 present the change in the electrode potential after the hydrogenation of alloys.

**Figure 10:**
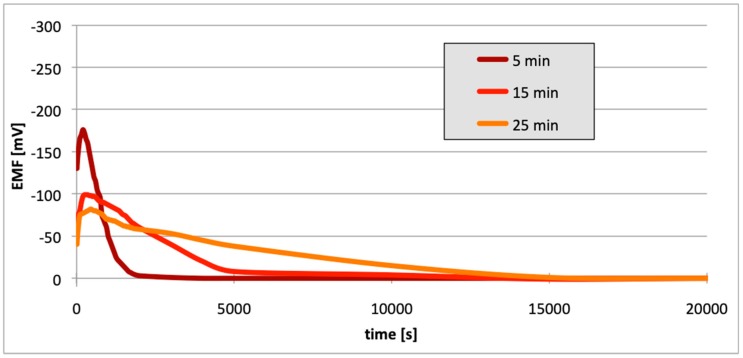
Decrease of cell EMF in time accompanying hydrogen desorption (EN AW-1050A). Hydrogenation time: 5, 15, and 25 min.

**Figure 11:**
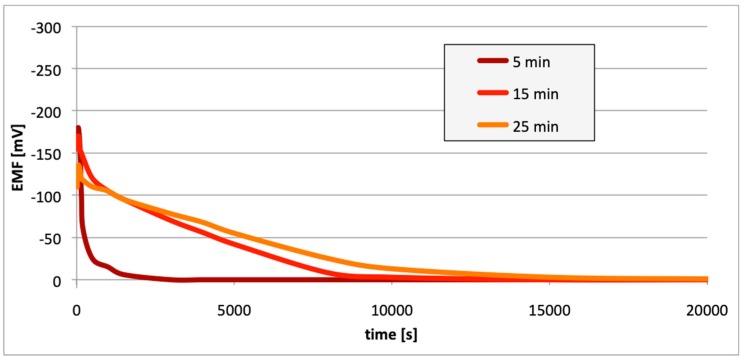
Decrease of cell EMF in time accompanying hydrogen desorption (EN AW-5754). Hydrogenation time: 5, 15, and 25 min.

**Figure 12:**
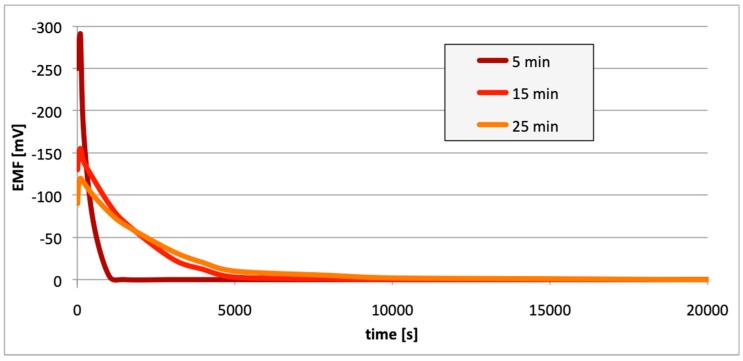
Decrease of cell EMF in time accompanying hydrogen desorption (EN AW-6060). Hydrogenation time: 5, 15, and 25 min.

Figure 10, Figure 11 and Figure 12 contain data with regard to the decrease in cell EMF over time during the desorption of hydrogen from one of the electrodes. The hydrogenation time of the samples prior to the measurements was equal at 5, 15, and 25 min.

**Figure 13:**
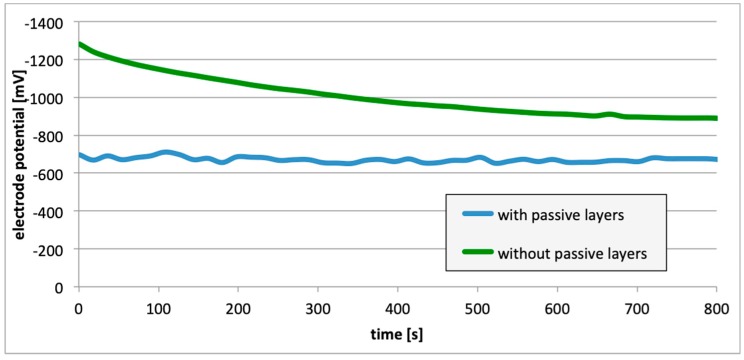
Electrode potential change following removal of passive layers (EN AW-1050A).

**Figure 14:**
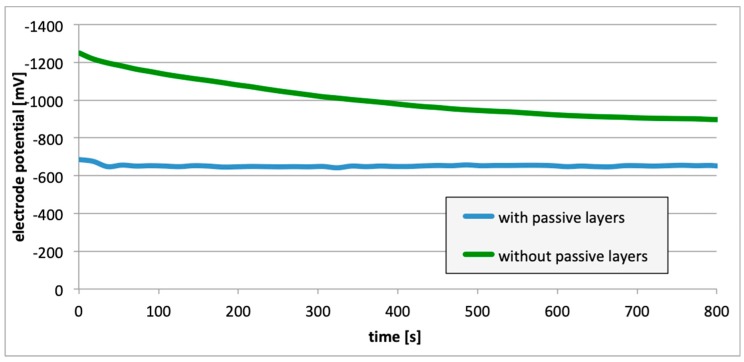
Electrode potential change following removal of passive layers (EN AW-5754).

**Figure 15:**
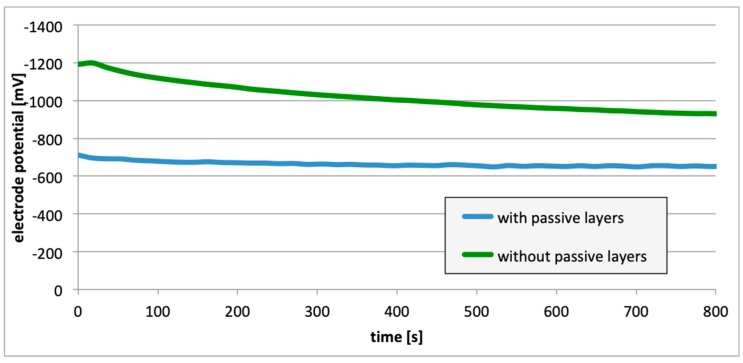
Electrode potential change following removal of passive layers (EN AW-6060).

Subsequently, potential measurements were carried out for the electrodes with the removed passive layer. Figure 13, Figure 14 and Figure 15 contain details regarding the variations in electrode potential (after removing the passive layers) to the negative side.

**Figure 16:**
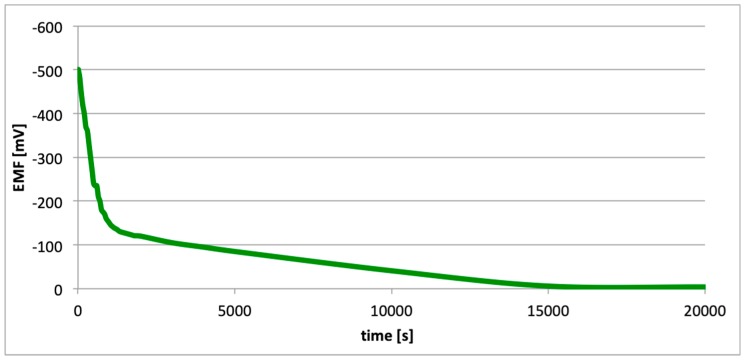
Decrease of cell EMF in time, accompanying absence of passive layer (EN AW-1050A).

**Figure 17:**
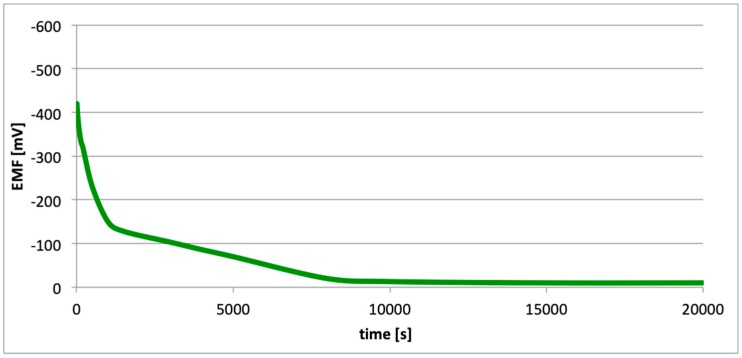
Decrease of cell EMF in time, accompanying absence of passive layer (EN AW-5754).

**Figure 18:**
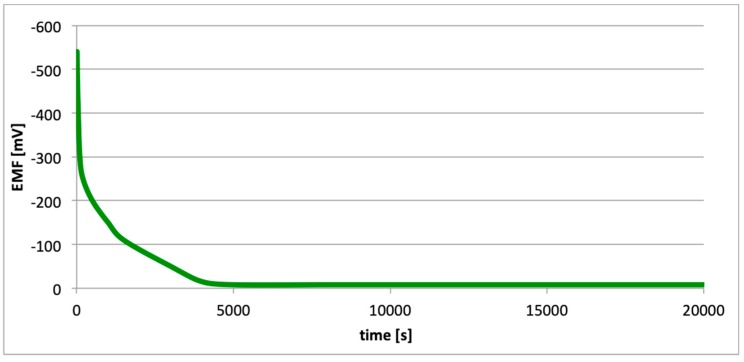
Decrease of cell EMF in time, accompanying absence of passive layer (EN AW-6060).

Figure 16, Figure 17 and Figure 18 contain data regarding the decrease of cell EMF accompanying the absence of a passive layer on one of the electrodes.

## 4. Discussion

In all cases (Figure 7, Figure 8 and Figure 9), following the hydrogenation of the alloy, the electrode potential shifted to the negative side (for AW-1050A alloy shifted of about −111 mV, for EN AW-5754 −58 mV, and for EN AW-6060 −57 mV), which clearly demonstrates that hydrogen affects the variations in the potential of the alloy surface. Thus, the hydrogenated alloy takes over the function of the anode. Therefore, further EMF measurements of the cell were performed (Figure 4). Measurements demonstrated that in each case a galvanic cell was created capable of generating EMF. EMF measurements of the cell after the hydrogenation of one of the electrodes demonstrated that this force decreases over time. This is due to hydrogen desorption during the measurements. Desorption time and level of EMF are closely related to the time of hydrogenation of the samples (Figure 10, Figure 11 and Figure 12). We can note that the shorter hydrogenation time leads to higher the initial EMF value of the cell (the highest initial values were recorded after 5 minutes of hydrogenation, for EN AW-1050A alloy −176 mV, for EN AW-5754 −180 mV and for EN AW-6060 −290 mV). However, longer hydrogenation time results in the longer life of the cell, despite the lower initial EMF value (Figure 10, Figure 11 and Figure 12, orange lines). This is due to the fact that during hydrogenation from electrolytes, hydrogen accumulates below the alloy surface. Coupled with the short hydrogenation time, hydrogen desorbs quickly from the sample. However, following a long hydrogenation time, it permeates into the deeper layers of the alloy and desorbs from it in the long run. 

**Figure 19:**
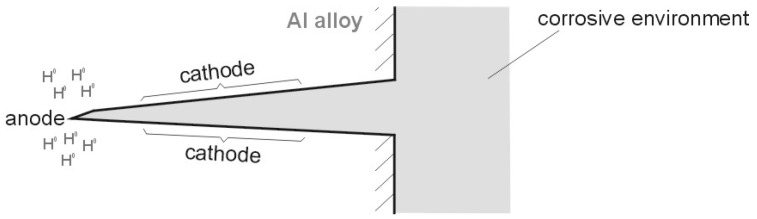
Corrosion cell formed in a crack following hydrogenation of an alloy.

However, regardless of the value of the cell EMF, this type of cell is generated e.g., in corrosion cracks (Figure 19). Since hydrogen accumulates in the tip of the crack (due to the largest tensile forces), this tip acts as an anode. In contrast, the walls (that do not contain hydrogen) perform the role of the cathode (Figure 19). The electrolyte in such a cell forms a corrosive environment, i.e., a moist environment of operation. Such a link is constantly closed, i.e., the load is the internal resistance of the alloy. During operation, such a link reduces the corrosive properties at the tip of the crack.

Such a cell acts like a hydrogen fuel cell and should be analyzed as such. The time of the operation of this type of cell results from the desorption time of hydrogen from the alloy. Quite evidently, from an energy point of view, the efficiency of such a cell is negligible (low amount of hydrogen, low reaction rate due to existence of aluminum electrodes/catalysts). Nevertheless, a constantly operating cell of this type can lead to the deterioration of the corrosive properties of aluminum alloys.

For measurements conducted in the conditions marked by the absence of the passive layer (as in the case of alloy hydrogenation), in all cases (Figure 13, Figure 14 and Figure 15), the electrode potential shifted to the negative (for EN AW-1050A alloy shifted of about −324 mV, for EN AW-5754 alloy −346 mV, and for EN AW-6060 −350 mV), which clearly demonstrates that the absence of the passive layer affects the alteration of the alloy surface potential. Therefore, further EMF measurements of the cell (Figure 4) were carried out while the passive layer was removed from one of the electrodes. 

**Figure 20:**
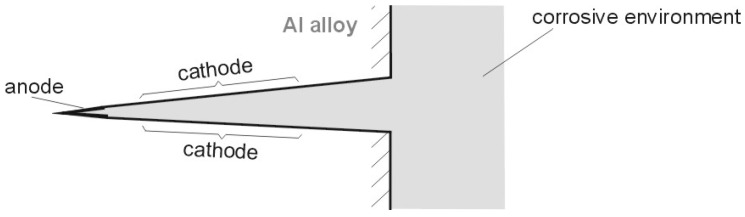
Corrosion cell originating in a crack after mechanical pitting of a crack.

The operating time of such a cell is synonymous with the time needed to restore the passive layer. We should note that the highest initial EMF value (−500 mV) was characterized by a cell with electrodes with the highest aluminum content (99.5%), i.e., the EN AW-1050A alloy (Figure 16). However, the lowest initial value of EMF (−540 mV) was characterized for the cell with electrodes made of the EN AW-6060 alloy (Figure 18). This alloy has the highest content of alloy elements (Table 1). Following the mechanical pitting (cracking) of the corrosion gap (e.g., as a result of exploitation of the structure comprising aluminum alloy), a surface absent of the passive layers is formed at the crack tip. This leads to the formation of a galvanic cell, in which in the absence of the passive layers, anode function is taken on by the pitting, and the walls of the crack act as anode (Figure 20). The electrolyte in such a cell is a corrosive environment, i.e., a moist operating environment. As in the case of hydrogen cells, such a cell is constantly closed, i.e., the load is the internal resistance of the alloy. During operation, such a link reduces the corrosion property at the tip of the crack (in the absence of passive layers).

## 5. Conclusions

This paper explored the effect of hydrogen and the absence of passive layers on the decrease of corrosive properties of aluminum alloys. The analysis involved structural aluminum alloys: EN AW-1050A, EN AW-5754, and EN AW-6060. It was demonstrated that the absorption of hydrogen by the analyzed alloys led to the shifted of the electrode potential to the negative side (Figure 7, Figure 8 and Figure 9). The developed and built hydrogen corrosion cells (Figure 4) demonstrate in each case the formation of EMF cells. The initial EMF value of the cell and its duration depends on the duration of hydrogenation (Figure 10, Figure 11 and Figure 12). As a result of removing the passive layers, the electrode potential also shifts to the negative side (Figure 13, Figure 14 and Figure 15). Following the removal of the passive layer from one of the electrodes, the cells also generated a galvanic (metal) cell. The duration of such a cell is equivalent to the time of restoration of the passive layer (Figure 16, Figure 17 and Figure 18). The formation of such hydrogen and metal galvanic cells changes the electrochemical properties of aluminum alloys, therefore deteriorates the corrosive properties of aluminum alloys (Figure 19 and Figure 20). Therefore, the process of designing elements made of aluminum alloys or installations related to the transport or production of hydrogen needs to take into account the level of hydrogen permeation in operating conditions.

## Figures and Tables

**Table 1:** Chemical composition of aluminum alloys applied in measurements [41].

Alloy Designation	Si	Fe	Cu	Mn	Mg	Cr	Zn	Ti	Others
EN AW-1050A	0.25	0.40	0.05	0.05	0.05	-	0.07	0.05	-
EN AW-5754	0.4	0.4	0.1	0.5	2.6–3.6	0.3	0.2	0.15	0.15
EN AW-6060	0.3–0.6	0.1–0.3	0.1	0.1	0.3–0.6	0.05	0.15	0.1	0.15
